# Epigallocatechin-3-gallate: A phytochemical as a promising drug candidate for the treatment of Parkinson’s disease

**DOI:** 10.3389/fphar.2022.977521

**Published:** 2022-09-12

**Authors:** Yumin Wang, Shuang Wu, Qiang Li, Weihong Lang, Wenjing Li, Xiaodong Jiang, Zhirong Wan, Jichao Chen, Hongquan Wang

**Affiliations:** ^1^ Department of Respiratory and Critical Care Medicine, Aerospace Center Hospital, Peking University Aerospace School of Clinical Medicine, Beijing, China; ^2^ Department of Neurology, Zhongnan Hospital of Wuhan University, Wuhan, China; ^3^ Department of Neurology, The Affiliated Hospital of Chifeng University, Chifeng, China; ^4^ Department of Psychological Medicine, The Affiliated Hospital of Chifeng University, Chifeng, China; ^5^ Department of Anatomy, College of Basic Medicine, Chifeng University Health Science Center, Chifeng, China; ^6^ Department of Neurology,Aerospace Center Hospital, Peking University Aerospace School of Clinical Medicine, Beijing, China; ^7^ Tianjin Medical University Cancer Institute and Hospital, National Clinical Research Center for Cancer, Tianjin’s Clinical Research Center for Cancer, Key Laboratory of Cancer Prevention and Therapy, Tianjin, China

**Keywords:** Parkinson’s disease, epigallocatechin 3-gallate, oxidative stress, apoptosis, neuroinflammation, α-synuclein

## Abstract

Epigallocatechin 3-gallate (EGCG), an abundant polyphenolic component derived from green tea extract, possesses versatile bioactivities that can combat many diseases. During the last decade, EGCG was shown to be effective in experimental models of Parkinson’s disease (PD). Several experimental studies have suggested that it has pleiotropic neuroprotective effects, which has enhanced the appeal of EGCG as a therapeutic strategy in PD. In this review, we compiled recent updates and knowledge of the molecular mechanisms underlying the neuroprotective effects of EGCG in PD. We focused on the effects of EGCG on apoptosis, oxidative stress, inflammation, ferroptosis, modulation of dopamine production, and the aggregation of α-synuclein. The review highlights the pharmacological features of EGCG and its therapeutic implications in PD. Taken together, the accumulated data indicate that EGCG is a promising neuroprotective compound for the treatment of PD.

## Introduction

Parkinson’s disease (PD) is the second most common neurodegenerative disease. It is characterized by motor and non-motor symptom (Postuma et al., 2015). The degeneration of dopaminergic neurons located in the substantia nigra pars compacta (SNpc) of the brainstem (Wang et al., 2021), which leads to the depletion of striatal dopamine levels (Meder et al., 2019), is the major pathological feature of PD, along with the presence of Lewy bodies (LBs), which mainly consist of misfolded α-synuclein, ubiquitin, PTEN-induced kinase-1 (PINK1), parkin, and other proteins, in the surviving neurons (Cookson, 2005; Abeliovich and Flint, 2006). PD affects more than 2% of the population older than 65 years old (Aarsland et al., 2017), and is becoming a major age-related health problem (Zou et al., 2015; Hirsch et al., 2016; Savica et al., 2016).

Despite intensive research, the molecular mechanisms involved in the degeneration of dopaminergic neurons remains poorly understood (Dos Santos et al., 2022). Oxidative stress (Fahn and Cohen, 1992; Dionísio et al., 2021), mitochondrial dysfunction (Park et al., 2018), neuroinflammation (Kip and Parr-Brownlie, 2022), iron dysregulation (Vuuren et al., 2020), ferroptosis (Ko et al., 2021; Mahoney-Sánchez et al., 2021; Wu et al., 2021), protein misfolding and degradation dysfunction (Haque et al., 2022), and environmental and genetic factors (Kline et al., 2021) probably play an important role in the pathogenesis of PD. The available therapeutic options for PD are limited, and only provide symptomatic relief, rather than halting the progression of the disease, in addition to having serious side effects (Wang et al., 2021). Increasing numbers of studies have been performed to identify neuroprotective compounds that can prevent dopaminergic neuron injury, and thereby retard disease progression and add further benefits to current therapy (Kujawska and Jodynis-Liebert, 2018; Dos Santos et al., 2022).

In this context, nutraceuticals have gained tremendous interest in recent decades, due to their long history of use (Payne et al., 2022). Various nutraceuticals exhibit antioxidative, anti-inflammatory, and anti-aging properties, and have been studied in the treatment of PD. Phytochemicals are biologically active nutraceutical plant chemicals that are typically secondary metabolites present in plants, such as green tea polyphenols, anthocyanidins, carotenoids, phytoestrogens, and terpenoids (Balakrishnan et al., 2021). Many phytochemicals have emerged as potential multi-target agents for the treatment of PD, due to their diverse actions (Peluso and Serafini, 2017).

Several dietary phytochemicals have been investigated in PD due to their potential beneficial and neuroprotective effects, including green tea catechins, such as epigallocatechin 3-gallate (EGCG) (Gonçalves et al., 2021). EGCG is an abundant polyphenolic component of green tea extract, and has exhibited versatile bioactivities in combating several diseases (Zhang et al., 2020; Fernandes et al., 2021). During the last decade, EGCG has been shown to be effective in experimental models of PD (Payne et al., 2022). Mounting evidence from experimental studies has suggested that EGCG exerts pleiotropic neuroprotective effects, which has led to emergence of EGCG as a therapeutic strategy for PD.

We here compiled recent updates on the use, and reports on the cellular and molecular mechanisms of neuroprotection of EGCG in PD. In this review, we focused on the effects of EGCG apoptosis, oxidation, inflammation, dopamine production, and the aggregation of α-synuclein. By highlighting the pharmacological features of EGCG and its therapeutic implications in PD, this review suggests that EGCG may be a promising neuroprotective compound for the treatment of PD.

### Source, biochemistry, and bioavailability of EGCG

**FIGURE 1:**
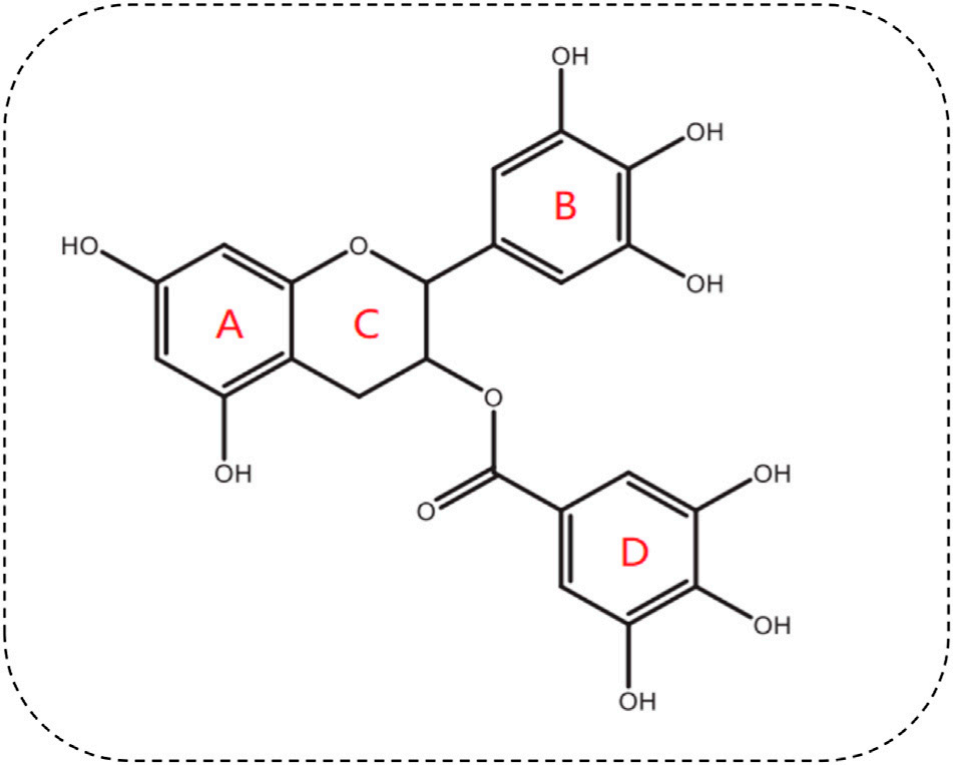
Chemical structure of EGCG.

Green tea contains six main catechin compounds, i.e., gallocatechin, catechin, epicatechin (EC), epicatechin gallate (ECG), epigallocatechin (EGC), and EGCG. EGCG is the most active component and best-studied polyphenol in green tea. Each two hundred and fifty milliliters (1.25% w/v) of green tea contains around 177 mg of EGCG (Alam et al., 2022). EGCG (C_22_H_18_O_11_) is a flavanol catechin, and is an ortho-benzoyl benzopyran byproduct, comprised of three hydroxyphenyl and hydroxybenzoate moieties marked A, B, C, and D (Payne et al., 2022) (Figure 1). The benzopyran ring, which has a phenyl group at C2 and a gallate group at C3, is made up of ring A and C. The B ring has positional 3,4,5-trihydroxyl groups, and the D ring gallate group (a galloyl moiety) is configured as an ester at C3. EGCG has reactive oxygen species (ROS)-deactivating properties due to the contribution of the B and D rings. The D ring has been shown to have anticancer and anti-inflammation characteristics (Payne et al., 2022). EGCG has seven hydroxyl radicals distributed among three aromatic rings, which confers water solubility, causing EGCG to have high blood-brain barrier (BBB) permeability (Payne et al., 2022). It has been reported that EGCG permeates the BBB within 0.5 h (Unno et al., 2017). The BBB permeability of EGCG were decreased by 57.54% (Unno et al., 2017). Although EGCG has good pharmacological and biological activity, the bioavailability of oral EGCG is relatively poor. A previous study showed that the highest plasma concentration of EGCG was only 0.15 µM after a human ingested two cups of green tea (Zhang et al., 2013). Oral EGCG was not stable in intestinal and blood environment, most of EGCG was not absorbed, and its bioavailability was reduced. The bioavailability of oral EGCG could be signifificantly improved through structure modification or nano-materials dependant protection and delivery (Dai et al., 2019).

**FIGURE 2:**
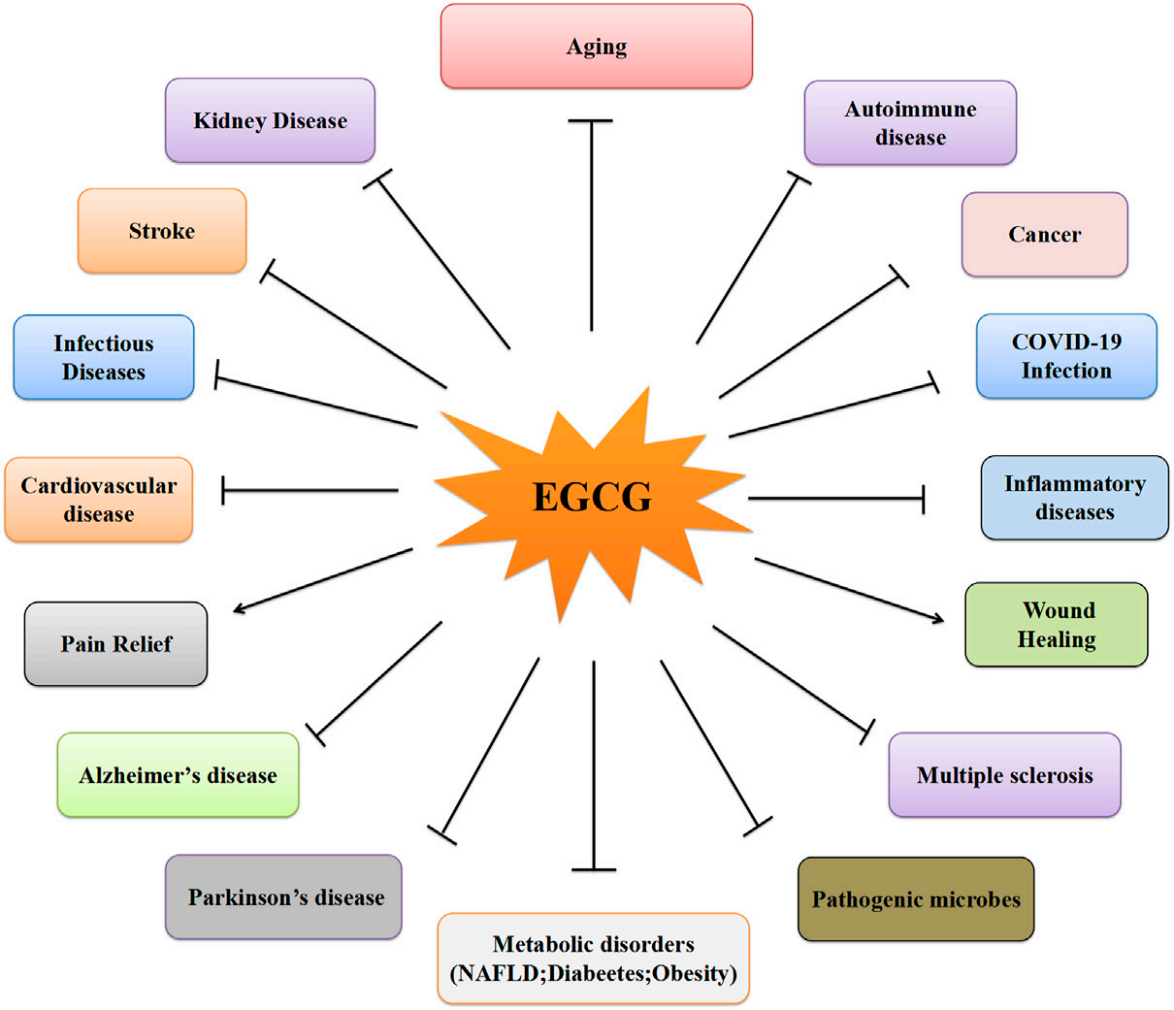
The different effects of EGCG on the different diseases.

The medicinal properties of green tea are derived from EGC esterification with gallic acid (i.e., galloylation). Thus, green tea has antioxidative mechanisms provided by EGCG (Payne et al., 2022). The unique chemical structure and makeup of EGCG confer its highly antioxidative and anti-inflammatory properties. EGCG is a peroxynitrite scavenger that reduces the nitration of tyrosine, and scavenges hydrogen peroxide and superoxide anions, thereby blocking ROS-induced DNA damage. EGCG have exhibited many disease-alleviating properties particularly regarding neuroprotective effects (Figure 2).

### Neuroprotective properties of EGCG in PD

During the last two decades, studies have increasingly focused on the neuroprotective properties of EGCG in PD. In the early 2000s, the potent neuroprotective effects of EGCG were studied in 6-hydroxydopamine (6-OHDA)-induced PC12 cells. These studies suggested that EGCG has neuroprotective effects against 6-OHDA-induced neuronal apoptosis (Jin et al., 2001). Since these first reports on the neuroprotective effects of EGCG in PD, EGCG has received significant attention as a therapeutic agent, due to its multiple molecular mechanisms of action in PD. The potential neuroprotective effects in the context of PD have been thoroughly studied in both *in vitro* and *in vivo* models, allowing a deeper understanding of the molecular cascades through which EGCG exerts its neuroprotective actions on PD (Table 1).

**TABLE 1:** Neuroprotective effects of EGCG in PD.

EGCG dose	Experimental model	Effects	Signaling	Involved mechanism	Ref
200 µM	6-OHDA/PC12 cell	↑Cell viability	-	-	Jin et al. (2001)
200 µM,pretreatment	6-OHDA/PC12 cell	↑Cell viability; ↓ Apoptosis	-	-	Nie et al. (2002a)
25–200 μM	6-OHDA/PC12 cell	↑Cell viability; ↓ Apoptosis	NA	Anti-apoptotic	Nie et al. (2002b)
0.1–10 μM,pretreatment	6-OHDA/SH-SY5Y	↑Cell viability; ↓ Apoptosis	↑pSTAT3	Anti-apoptotic	Wang et al. (2009)
100 μM	6-OHDA/A53T-α-syn SH-SY5Y cell	↑Cell viability; ↓ Apoptosis;	-	Anti-apoptotic	Ma et al. (2010)
10 μM	6-OHDA/SH-SY5Y	↑Cell viability; ↓ LDH	↑Akt	-	Chao et al. (2010)
1–10 mg/kg	6-OHDA/N27 cell	↑Cell viability; ↓caspase-3; ↓ DMT1; ↓hepcidin; ↑FPN1; ↓Fe^2+^; ↑TH^+^ primary mesencephalic neurons	-	Anti-apoptotic	Chen et al. (2015)
10 mg/kg	6-OHDA/Male Wistar rats	↓Rotational behavior; ↑locomotor activity; ↑antidepressive effects; ↑cognitive dysfunction; ↓oxidative stress	-	Antioxidant	Bitu Pinto et al. (2015)
100 μM	6-OHDA/SK-N-AS	↑Cell viability; ↓caspase-3; ↓IL-1β and TNF-α	-	Anti-apoptotic; Anti-inflammatory	Özduran et al. (2022)
1–10 μM	DDT/SH-SY5Y	↑Cell viability	-	-	Tai and Truong, (2010)
10–30 μM	Glutamate/HT22 cell	↓L-DOPA methylation; ↓NF-kB; ↓ROS; ↓GFAP-immunoreactive astrocytes (CA3 region);		Antioxidant COMT inhibitor	Kang et al. (2010)
100 mg/kg	L-DOPA/rat	↑Striatal dopamine; ↓3-OMD level	-	COMT inhibiton	Kang et al. (2010)
30 min before i.c.v. injection of kainic acid	Kainic acid/rat	↓Oxidative stress	-	Antioxidant; COMT inhibitor	Kang et al. (2010)
100 μM	L-DOPA/PC12 cell	↑Cell viability; ↑GSH	-	Antioxidant	Lee et al. (2013)
100 μM	LPS/Primary Microglia	↓NO release; ↓TNF-α; ↓iNOS	-	Anti-inflammatory	Li et al. (2004)
100 μM	LPS/SH-SY5Y	↑Cell survival	-	-	Li et al. (2004)
200–400 μM	LPS/SD rat	↓NO; ↓TNF-α; ↓iNOS; ↑Striatal dopamine; ↑TH^+^ neurons in midbrain	-	-	Al-Amri et al. (2013)
EGCG-Loaded Liposomes	LPS/BV-2 microglia	↑Cell survival; ↓oxidative stress; ↓NO; ↓TNF-α; ↓cPLA2; ↓COX-2	-	Antioxidant; Anti-inflammatory	Cheng et al. (2021)
EGCG-Loaded Liposomes	LPS/SD rat	Restored motor impairment; ↓NO release; ↓TNF-α; ↓IL-1β	-	Anti-inflammatory	Cheng et al. (2021)
1.25–10 μM	MPP+/PC12 cell	↑Cell survival; ↓ROS; ↑SIRT1; ↑PGC-1α, SOD1 and GPX1	↑SIRT1/PGC-1α	Antioxidant	Ye et al. (2012)
Lep/RES-EGCG-liposomes	MPP+/SH-SY5Y	↑Cell survival; ↑Bcl-2; ↓Bax; ↓α-syn; ↑TH; ↑dopamine transporter	-	Anti-apoptotic	Kuo et al. (2021)
25 mg/kg (p.o.)	MPTP/mice	↑TH-positive cells (SN); ↑TH activity (striatum); ↑ dopamine (striatum); ↑HVA; ↓nNOS (SN)	-	-	Choi et al. (2002)
50 mg/kg	MPTP/mice	↑TH-positive cells in the substantia nigra; ↓iNOS	-	-	Kim et al. (2010)
25 mg/kg, 7 d	MPTP/mice	↓Rotational latency; ↑striatal levels of dopamine; ↓oxidative stress; ↑DOPAC; ↑ferroportin	-	Antioxidant	Xu et al. (2017)
25–50 mg/kg/day	MPTP/mice	↓Motor dysfunction; ↑TH-positive cells in the substantia nigra; ↓TNF-α; ↓IL-6; ↑CD3+CD4^+^ to CD3^+^CD8^+^ T lymphocytes in the peripheral blood	-	Modulating peripheral immune response	Zhou et al. (2018)
1–200 μM	Paraquat/PC12 cell	↑Cell survival; ↑mitochondrial membrane potential; ↓ caspase-3; ↓ pro-apoptotic protein Smac in cytosol	-	Anti-apoptotic	Hou et al. (2008)
0.1–0.5 mM	Paraquat/knock-down parkin *Drosophila melanogaster*	↑Life span and locomotor activity; ↓oxidative stress	-	Antioxidant	Bonilla-Ramirez et al. (2013)
0.5 mM	Paraquat/knock-down parkin *Drosophila melanogaster*	↑Life-span; ↑locomotor activity; ↓LPO; ↓neurodegeneration	-	Antioxidant	Martinez-Perez et al. (2018)
100 or 300 mg/kg i.p	Rotenone/Male SD rats	↓Motor Impairment; ↓NO; ↓LPO; ↑GSH, SOD, and CAT; SDH, total ATPase, NADH cytochrome C reductase, and succinate-cytochrome C reductase; ↓TNF-α; ↓IL-1β; ↓IL-6; caspase-3	-	Antioxidant Anti-apoptotic Anti-inflammatory	Tseng et al. (2020)
20 μM	-	Convert large, mature α-synuclein and amyloid-β fibrils into smaller, amorphous protein aggregates	-	Disassembles preformed amyloid fibrils	Bieschke et al. (2010)
100 nM	-	↓α-syn aggregation	-	-	Xu et al. (2016)
20 μM	-	↓α-syn fibril	-	-	Jha et al. (2017)
20 μM	α-syn/SH-SY5Y	↑Cell survival; ↓LDH	-	-	Jha et al. (2017)
20 μM	-	↓α-syn fibril	-	-	Zhao et al. (2017)
10 μM	α-syn/PC12	↑Cell survival; ↓ROS		Antioxidant	Zhao et al. (2017)
10–70 μM	α-syn/SH-SY5Y	↓α-syn-mediated cytotoxicity	-	-	Yang et al. (2017)
20 mM	α-syn transduced-PC12 cells	↑Cell viability; ↓Cu(II) induced fibrillation of α-syn; ↓α-syn overexpression	-	-	Teng et al. (2019)
5–50 μM	-	Disaggregates the protofibrils and mature γ-syn fibrils into similar SDS resistant oligomers	-	-	Roy and Bhat, (2019)
50 μM	γ-syn oligomers/SH-SY5Y	↑Cell survival; ↓LDH	-	-	Roy and Bhat, (2019)
Molar ratio of EGCG to α-syn is 2:1	-	Destabilizes α-synuclein fibril; disrupts the β-sheet structures of α-syn fibril		-	Yao et al. (2020)
EGCG homogenous microparticles 30 μM	α-syn oligomers/N2A cell	Inhibited the amyloidogenic aggregation of α-syn cytotoxic effects of α-syn oligomers; ↑Cell survival; ↓LDH	-	-	Fernandes et al. (2020)
0.1–0.5 mM	*Drosophila melanogaster* with PINK1 mutations	↓Locomotive and neuronal defects; remodeling gut microbiota	-	-	Xu et al. (2020)
0.5 mM	LRRK2 and parkin-null flies	↑Climbing scores in EGCG-treated mutant LRRK2 flies; ↓loss of DA neurons displayed by Ddc GAL4-LRRK2 G2019S-expressing flies; ↓enlarged mitochondria in their DA neurons	-	-	Ng et al. (2012)

↑, indicates upregulation; ↓, indicates downregulation; DMT1, divalent metal transporter-1; Fpn1, ferroportin 1; DDT, dichlorodiphenyl-trichloroethane; 3-OMD, 3-O-methyldopa; LPS, Lipopolysaccharide; NO, nitric oxide; iNOS, inducible NO, synthase; Lep/RES-EGCG-liposomes,leptin-conjugated phosphatidic acid liposomes with resveratrol and epigallocatechin gallate; HVA, 3,4-dihydroxyphenylacetic acid and homovanillic acid; PINK1, PTEN induced putative kinase 1; TH, tyrosine hydroxylase; LPO, lipid peroxidation; SN, substantia nigra.

### Protection against apoptosis

Apoptosis is activated via the intrinsic or extrinsic pathways, and has been extensively documented in PD (Stern, 1996; Dionísio et al., 2021). Apoptosis has been implicated as the main mechanisms of neuronal death in the SNpc in PD. Apoptotic cell death has been observed in cell culture and animal models of PD, and also in nigrostriatal regions of the brains of patients with PD at postmortem (Lev et al., 2003). Targeting apoptosis is regarded as one strategy for preventing dopaminergic neuron death (Vila et al., 2001; Ma et al., 2016).

Jin et al.’s pioneering study showed that preincubation with EGCG inhibited 6-OHDA-induced apoptosis in PC12 cells (Jin et al., 2001), which was further corroborated by the same group’s later studies (Nie et al., 2002a; 2002b). After these studies, evidence suggesting that EGCG exerts neuroprotective effects against apoptosis in PD has accumulated. Levites and others have shown that EGCG prevented both 6-OHDA-induced expression of several mRNAs, such as *Bad*, *Bax*, and *Mdm2*, and resulted in a decrease in Bcl-w, Bcl-2, and Bcl-x(L). EGCG exerted neuroprotective effects against 6-OHDA caused SH-SY5Y cells toxicity through increasing phosphorylated protein kinase C (PKC), suggesting that EGCG exert neuroprotective effects against oxidative stress-induced cell death through activation of PKC and modulation of apoptosis (Levites et al., 2002). Chan and others have shown that pretreatment of SH-SY5Y cells with EGCG at 0.1–10 μM significantly attenuated cell death induced by 6-OHDA. EGCG (1 μM) prevented 6-OHDA-induced activity decline of STAT3. These data clearly demonstrated that EGCG inhibited 6-OHDA-induced oxidative stress-dependent cell death through re-stimulation of the STAT3 signaling pathway (Wang et al., 2009). EGCG inhibited 6-OHDA-induced neurotoxicity in SH-SY5Y cells expressing A53T-mutated α-synuclein, by which sensitivity to 6-OHDA was increased, causing oxidative stress (Ma et al., 2010). EGCG protected against 6-OHDA-induced neurotoxicity in N27 cells. Pretreatment with EGCG prevented the 6-OHDA-induced activation of caspase-3 activity (Chen et al., 2015). In the 6-OHDA-treated SK-N-AS cell PD model, EGCG inhibited the upregulation of α-synuclein, and significantly reduced caspase-3 immunoreactivity (Özduran et al., 2022). A recent study has suggested that leptin-conjugated phosphatidic acid liposomes containing EGCG and resveratrol reduced 1-methyl-4-phenylpyridinium (MPP^+^)-induced apoptosis in SH-SY5Y cells (Kuo et al., 2021). EGCG and resveratrol, encapsulated in liposomes, could reduce expression of Bax and α-synuclein, and increase levels of Bcl-2, tyrosine hydroxylase (TH), and the dopamine transporter (Kuo et al., 2021). EGCG also inhibited apoptosis induced by paraquat (PQ) in PC12 cells (Hou et al., 2008), by inhibiting the loss of mitochondrial membrane potential (MMP) as well as reducing caspase-3 activity, and by downregulating levels of the pro-apoptotic protein Smac in the cytosol (Hou et al., 2008). Furthermore, EGCG inhibited apoptosis induced by rotenone *in vivo* (Tseng et al., 2020). In rotenone-challenged rat PD models, EGCG treatment prevented most of the rotenone-induced motor dysfunctions. EGCG reduced the levels of the apoptotic marker caspase-3 in the striatum of these rats (Tseng et al., 2020). Taken together, EGCG shows potential in inhibiting apoptosis in both *in vivo* and *in vitro* PD models.

### Protection against oxidative stress

Oxidative stress is one of the main factors in the pathogenesis of PD (Percário et al., 2020; Dorszewska et al., 2021). The oxidative stress hypothesis of PD was proposed in 1992 (Fahn and Cohen, 1992), and holds that oxidative stress leads to the neurodegeneration of dopaminergic neurons, resulting in the pathogenesis of PD (Subramaniam and Chesselet, 2013). Accumulating evidence has suggested a number of sources and mechanisms for oxidative stress in PD, which include nicotinamide adenine dinucleotide phosphate oxidase (NOX) activation, mitochondrial dysfunction, the catabolism of dopamine by auto-oxidation, iron (Fe^2+^) accumulation (Wang et al., 2021). Oxidative stress causes injury to macromolecular components (i.e., DNA, proteins, and lipids) (Pires et al., 2019a; 2019b; 2019c; 2019d, 2020), resulting in cellular dysfunction and, eventually, dopaminergic neuron death (Wang et al., 2021). Given the important role of oxidative stress in PD, antioxidant supplements could be a reasonable therapeutic approach to halting PD progression (Chang and Chen, 2020), as it could mitigate oxidative stress-dependent neuronal injury (Buendia et al., 2016).

Ye et al. (2012) and Lee et al. (2013) highlighted the EGCG-mediated decrease in PD-related neurotoxin-induced ROS production in their *in vitro* experiments. Ye et al. showed that EGCG inhibits MPP^+^-induced oxidative stress in PC12 cells via the SIRT1/PGC-1α signaling pathway (Ye et al., 2012). Specifically, EGCG significantly increased cell viability and decreased MPP^+^-induced ROS production, and potentiated MPP^+^-induced upregulation of Sirtuin 1 (SIRT1), peroxisome proliferator-activated receptor gamma (PPARgamma) coactivator-1α(PGC-1α), glutathione peroxidase (GPX1), and superoxide dismutase 1 (SOD1) (Ye et al., 2012). Lee et al. demonstrated that EGCG could inhibit L-3,4-dihydroxyphenylalanine-induced oxidative stress-dependent PC12 cell death, which was reflected by a reduction in ROS generation and production of thiobarbituric acid reactive substances, and by an increased intracellular level of glutathione (GSH) (Lee et al., 2013).

EGCG also plays a neuroprotective role in PD through antioxidant mechanisms *in vivo* PD animal models. In a 1-methyl-4-phenyl-1,2,3,6 -tetrahydropyridine (MPTP)-induced PD model, EGCG rescued MPTP-induced neurotoxicity by decreasing serum protein carbonyls, implying that EGCG reduced oxidative stress in mice (Xu et al., 2017). In agreement with these findings, Pinto and others revealed that EGCG reverted behavioral changes in 6-OHDA-induced male Wistar rats, which were reflected by increased locomotor activity, decreased rotational behavior, antidepressive effects, and improvement of cognitive dysfunction. EGCG reversed the striatal oxidative stress and inhibited immunohistochemistry changes, indicating that EGCG likely exerts neuroprotective effects by its powerful antioxidant and anti-inflammatory properties (Bitu Pinto et al., 2015). This observation was corroborated by other studies, which reported that EGCG protects and prevents PQ-induced oxidative stress-dependent neurodegeneration in *Drosophila melanogaster* (Bonilla-Ramirez et al., 2013; Martinez-Perez et al., 2018). Recent evidence has indicated that EGCG reversed rotenone-induced neurochemical and motor dysfunctions in rats by reducing lipid peroxidation (LPO) and nitric oxide (NO) levels (Tseng et al., 2020). This study substantiated previous indications that EGCG had neuroprotective effects in PD by anti-oxidant, anti-neuroinflammation, and anti-apoptosis activities (Tseng et al., 2020). Taken together, EGCG shows potential in inhibiting neurotoxin-induced oxidative stress injury in both *in vitro* and *in vivo* PD models.

### Protection against neuroinflammation

Since McGeer and others observed activated microglial infiltration in the SN of the postmortem PD brain, in the early 1980s (McGeer et al., 1988), numerous studies have focused on the role played by neuroinflammation in the pathogenesis of PD. These studies have revealed that cytokine-induced inflammatory responses play an important role in this disease (Liu et al., 2022). Activation of astrocytes/microglia and peripheral immune cell infiltration, a process called neuroinflammation, are observed in PD (Kip and Parr-Brownlie, 2022). Chronic inflammation and neuroinflammation triggers neuronal damage and plays a vital role in PD pathology (Tansey and Romero-Ramos, 2019; Pajares et al., 2020; Yang et al., 2020; Badanjak et al., 2021; Hirsch and Standaert, 2021; Kip and Parr-Brownlie, 2022). Mounting evidence has indicated that targeting chronic inflammation may be a potential therapeutic target for PD, and pharmacologically reducing neuroinflammation via therapeutic compounds maybe prevent or delay progression of PD (Wang et al., 2015; Hassanzadeh and Rahimmi, 2018; Lee et al., 2019, 2021; Kip and Parr-Brownlie, 2022).

Remarkably, EGCG exhibits anti-inflammatory activities *in vitro*. Le and others have shown that EGCG potently down-regulates inducible NO synthase (iNOS) and tumor necrosis factor-α (TNF-α) expression, thereby inhibiting lipopolysaccharide (LPS)-activated microglial secretion of nitric oxide (NO) and TNF-α. In addition, EGCG inhibited neuronal injury in SH-SY5Y and in primary rat mesencephalic cultures through microglial activation, which suggested that EGCG functions as a potent inhibitor of microglial activation, thereby alleviating microglia-mediated dopaminergic neuron injury in PD (Li et al., 2004). Additionally, EGCG suppresses 6-OHDA-induced expression of TNF-α and IL-1β in SK-N-AS cells, thereby inhibiting apoptotic pathways and enhancing survival (Özduran et al., 2022).

Recently, several *in vivo* findings have provided evidence for possible anti-inflammatory effects of EGCG in PD. Al-Amri et al. reported that pretreatment with EGCG decreased TNF-α and NO, and markedly increased the number and density of TH-immunoreactive neurons in the midbrain of PD model rats (Al-Amri et al., 2013). Likewise, EGCG reduced the rotenone-induced increase in NO levels in the striatum and reduced the levels of neuroinflammatory markers of model rats (Tseng et al., 2020). Interestingly, recent data have demonstrated that EGCG-loaded liposomes decreased the production of NO and TNF-α in LPS-induced BV-2 microglia, attenuated LPS-induced pro-inflammatory cytokine levels, and restored motor impairment *in vivo* in a PD rat model, suggesting that EGCG exerts a neuroprotective effect by modulating microglial activation (Cheng et al., 2021).

Collectively, these data indicate that EGCG maybe play a neuroprotective role by inhibiting neuroinflammation in both *in vivo* and *in vitro* PD models.

### Protection against ferroptosis

**FIGURE 3:**
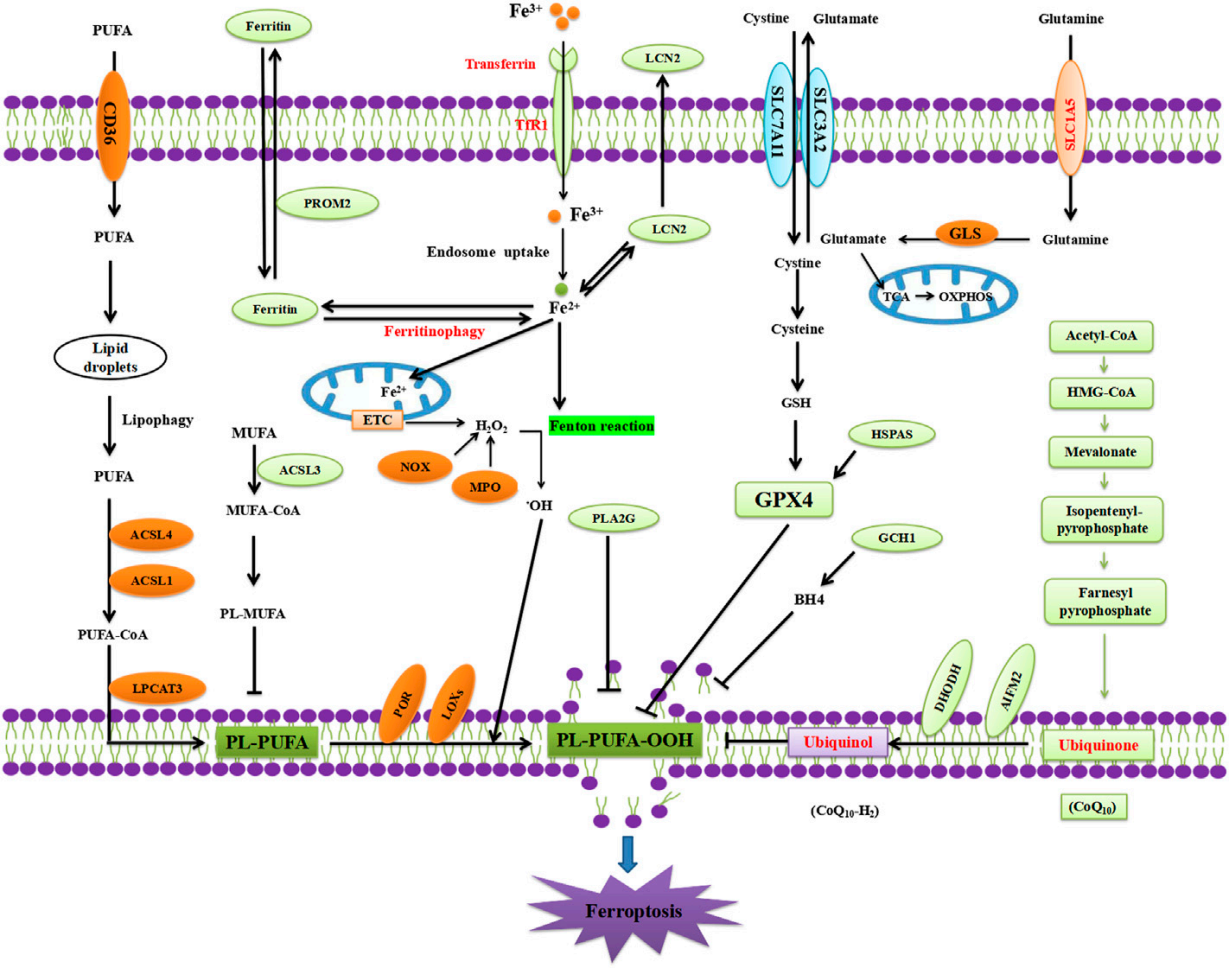
Mechanism of ferroptosis.

Recent studies have suggested that EGCG may regulate ferroptosis, which is an iron-dependent regulated cell death pathway involving a lethal accumulation of lipid peroxides that is triggered by a combination of iron toxicity, LPO, and plasma membrane damage (Zheng and Conrad, 2020; Chen et al., 2021; Stockwell, 2022) (Figure 3). Ferroptosis, characterised by iron-dependent LPO, shares several features with PD pathophysiology. Interestingly, several major pathological hallmarks of PD are known key features and/or triggers in the ferroptosis pathway (Mahoney-Sánchez et al., 2021). These include iron overload (Dexter et al., 1987), increased LPO (Dexter et al., 1986; de Farias et al., 2016), SLC7A11 downregulation (Vallerga et al., 2020), DJ-1 depletion (Cao et al., 2020), GSH level reduction (Sofic et al., 1992; Sian et al., 1994), and CoQ10 level reduction (Battino et al., 1996; Mischley et al., 2012). Increasingly, studies have revealed that α-synuclein regulates both iron and lipid metabolism, suggesting a possible interplay between ferroptosis and dysregulated α-synuclein (Angelova et al., 2020). Taken together, these studies strongly implicate ferroptosis in the neurodegeneration observed in PD.

Reddy and coworkers have shown that EGCG can affect brain iron homeostasis in 6-OHDA-induced N27 cells (Chen et al., 2015). EGCG pretreatment counteracted 6-OHDA-induced increased expression of divalent metal transporter-1 (DMT1) and hepcidin and decreased expression of the iron-export protein ferroportin 1 (Fpn1), leading to a 28% reduction in Fe^2+^ uptake. Pretreatment with EGCG prevented the 6-OHDA-induced activation of caspase-3 activity, indicating that EGCG inhibits 6-OHDA-induced neurotoxicity by regulating iron homeostasis (Chen et al., 2015). This observation was corroborated by other studies, which showed that EGCG upregulated Fpn1 in the SN and reduced oxidative stress, thereby exerting a neuroprotective effect against MPTP-induced neurotoxicity in mice (Xu et al., 2017). However, the study by Lee et al. (2013) demonstrated that EGCG increased intracellular levels of GSH in a PD model. Recent evidence indicated that EGCG reversed rotenone-induced lipid peroxidation (LPO) production (Tseng et al., 2020), which substantiate previous indications showing that EGCG treatment provided protection and prevention from the PQ-induced increase in LPO and neurodegeneration in dj-1-β-knockdown *Drosophila melanogaster* (Martinez-Perez et al., 2018).

In summary, these studies suggested that EGCG inhibits iron overload, decreased LPO, and increased GSH levels in PD models, which are the three major hallmarks of ferroptosis. However, further research is needed to strengthen this hypothesis and provide more detailed mechanisms underlining EGCG inhibition of ferroptosis, such as whether EGCG regulates the ferroptosis signaling pathway and ferroptosis regulators.

### Modulation of dopamine production

PD is a neurodegenerative disease caused by the death of dopaminergic neurons located in the SNpc of the brainstem, resulting in the depletion of striatal dopamine, an important neurotransmitter in the brain (Meder et al., 2019). Loss of more than 80% of the dopaminergic neurons in the SNpc affects the nigrostriatal circuits in the midbrain, leading to typical PD motor symptoms, which include tremor at rest, rigidity, slowness or absence of voluntary movement, postural instability, and freezing (Masato et al., 2019; Latif et al., 2021). The recovery of striatal DA content is an important target in PD treatment. Therefore, dopamine replacement therapy, compensating for the lack of dopamine, is the classic treatment for motor symptoms of PD (Ferrazzoli et al., 2016).

A previous study revealed that EGCG (400 mg/kg) protected against MPTP-induced functional and neurochemical deficits, resulting in increased striatal dopamine concentrations in an MPTP-induced PD model in male C57 black mice (Xu et al., 2017). A single intraperitoneal injection of LPS (15 mg/kg) resulted in a decrease in dopamine levels and reduced the number and the density of TH-positive neurons in the midbrain in male Sprague–Dawley rats. Pretreatment with EGCG (10 mg/kg) preserved the number of TH-positive neurons and increased dopamine levels, indicating that EGCG protected against LPS-induced neurotoxicity by reducing inflammatory mediators and preserving dopamine levels in the midbrain (Al-Amri et al., 2013).

Two important enzymes, monoamine oxidase (MAO) and catechol-O-methyl transferase (COMT), are needed for the catabolism of dopamine, through which dopamine is changed to its inactive metabolites (Latif et al., 2021). MAO first converts dopamine to 3,4-dihydroxyphenylacetaldehyde (DOPAL). Aldehyde dehydrogenase then converts DOPAL to 3,4-dihydroxyphenylacetic acid (DOPAC). In the COMT pathway, dopamine is converted to 3-methoxytyramine, which is further reduced to homovanillic acid (HVA), which is subsequently eliminated via the urine (Latif et al., 2021). In the MPTP-induced PD murine model, EGCG inhibits the loss of TH-positive cells located in the SN and the reduction of TH activity in the striatum. At the same time, EGCG preserves dopamine and its metabolites, DOPAC and HVA, in the striatum (Choi et al., 2002).

### Modulation of α-Synuclein

The pathological hallmarks of PD are the presence of LBs in different brain regions, which are primarily composed of misfolded and aggregated α-synuclein (Singh et al., 2017). Increasing evidence has indicated that α-synuclein plays a pivotal role in PD pathogenesis. It has been reported that α-synuclein aggregation is one of the leading causes for dopaminergic neuron dysfunction and death (Srinivasan et al., 2021). The multifactorial events involved in this process includes increased oxidative stress, inflammation, mitochondrial dysfunction, and ubiquitin-proteasome system (UPS) dysfunction, which lead to the accumulation of abnormal or misfolded α-synuclein (Javed et al., 2018). These aggregates undergo several key stages of oligomerization, fibrillation, and aggregation. Recent studies have proposed that α-synuclein aggregates can disrupt synaptic regulation, impair neuronal signaling, and eventually lead to neuronal death (Volpicelli-Daley et al., 2011; Zoey et al., 2021). The α-synuclein oligomers induce mitochondrial dysfunction and cause neuroinflammation, oxidative stress, endoplasmic reticulum stress, and inhibition of proteasomal activity and autophagy (Ghiglieri et al., 2018; Javed et al., 2018). An imbalance in the homeostasis of α-synuclein might result in accumulation of α-synuclein and aggregation. The α-synuclein oligomer hypothesis of PD for dopaminergic neuron cell death holds that α-synuclein forms transiently unstable oligomers, which exert cytotoxic effects and are eventually converted to thermodynamically more stable amyloid fibrils (Gadhe et al., 2022).

EGCG inhibits α-synuclein fibrillogenesis in cell-free assays (Bieschke et al., 2010). After this was published, many studies investigated whether EGCG has the ability to remodel α-synuclein aggregates in cell-based models, and found that EGCG could reduce α-synuclein fibril-induced cytotoxicity by remodeling the α-synuclein structure (Bieschke et al., 2010). EGCG binds to α-synuclein amyloid fibrils and oligomers, thereby directly altering their morphology. It as shown that EGCG directly binds to β-sheet-rich aggregates, mediating a conformational change without disassembling them into small diffusible oligomers or monomers (Bieschke et al., 2010). Subsequently, it was shown that EGCG can robustly disaggregate pre-formed oligomers and dose-dependently inhibit α-synuclein aggregation (Caruana et al., 2011). Another study revealed that EGCG can reduce the ability of oligomers to bind to membranes, in addition to affecting oligomer size distribution or secondary structure, to prevent cytotoxicity (Lorenzen et al., 2014).

Jha and coworkers have shown that high dose EGCG decreased fibrillization kinetics, and concentration-dependently reduced the toxicity of α-synuclein aggregates. EGCG induced nontoxic aggregates to form smaller sized fibrils, indicating that EGCG may decrease α-synuclein aggregate-induced cytotoxicity by its ability to reduce the exposure of a hydrophobic surface (Jha et al., 2017).

Taken together, these studies suggested that EGCG have the poteential to protect against α-synuclein-induced cytotoxicity by modulating the α-synuclein aggregation pathway toward formation of nontoxic aggregates. Moreover, EGCG ameliorates cytotoxicity induced by α-synuclein oligomers, possibly by reducing the extent of toxic aggregate-induced cell membrane permeabilization.

## Future prospective and challenges

There are still some challenges on EGCG new drug development regarding to PD. The first challenge is that the stability of EGCG is poor, the absorption rate is low, the bioavailability of oral EGCG was relatively poor needs to be improved (Dai et al., 2019). Another challenge is BBB penetration property of EGCG (Zhang et al., 2020).

However, some authors have suggested new techniques to improve the bioavailability of EGCG, such as nanoparticle-based delivery systems, structurally modified molecules of catechins, or co-administration with other drugs or bioactive compounds (Cai et al., 2018; Gonçalves et al., 2021). Simultaneously, the precise molecular mechanism underlying the action of EGCG is not fully understood. The detailed cell signaling pathway through which EGCG exerts its neuroprotective effects require further investigation. Mechanistic research that can help to define the function of EGCG could provide further benefits for human health. To date, reliable clinical data describing the neuroprotective effects of EGCG for the treatment of PD are lacking. However, the beneficial effect of EGCG in PD still needs to be confirmed in larger animals or even in humans before they are applied in clinical settings. Hence, these aspects of EGCG need to be studied in future, and clinical trials on its efficacy and safety should be performed. EGCG remains a potential and promising therapeutic strategy in the battle against PD.

## Conclusion

**FIGURE 4:**
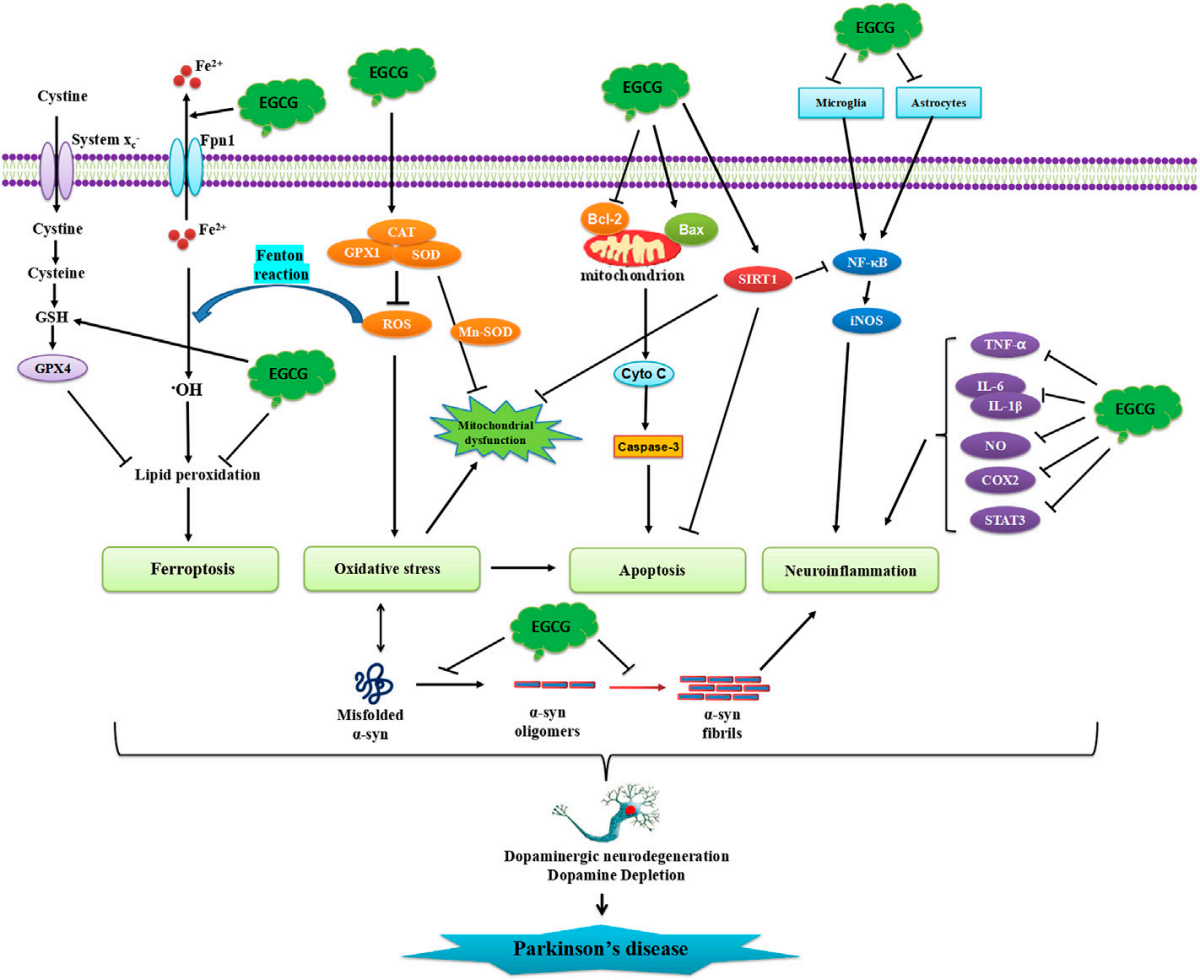
Schematic illustration of neuroprotective effects of EGCG in PD. EGCG can attenuate α-synuclein aggregation, oligomerization, and fibrillation. EGCG can also inhibit protein misfolding, oxidative stress, neuronal apoptosis, and neuroinflammatory responses.

In conclusion, we here summarized the neuroprotective roles of EGCG shown in both *in vitro* and *in vivo* PD models. The studies summarized in this review clearly revealed that EGCG may have the potential to be a novel drug for the treatment of PD, to prevent neurodegeneration due to its multi-targeted actions. The published research suggests that the molecular mechanisms by which EGCG exerts neuroprotective benefits include inhibition of apoptosis, oxidative stress, inflammation, and ferroptosis, modulation of dopamine production, and the aggregation of α-synuclein (Figure 4).
